# The influence of teaching motivation of female physical education teachers in basic education in China on job burnout: the mediating role of professional identity

**DOI:** 10.3389/fpubh.2026.1758736

**Published:** 2026-04-21

**Authors:** Yanni Liu, Sanming Fan, Yan Tang

**Affiliations:** 1 School of Physical Education, Shanghai University of Sport, Shanghai, China; 2 School of Physical Education, Shanghai Normal University, Shanghai, China

**Keywords:** female, job burnout, motivation for teaching, PE teachers, professional identity

## Abstract

**Objective:**

As professionals entrusted with delivering public education services, teachers are particularly susceptible to job burnout, which is shaped by a combination of individual and environmental factors. This study investigates the mechanisms through which teaching motivation and professional identity influence job burnout among female PE teachers, aiming to provide empirical evidence and actionable recommendations for refining PE policies and enhancing teacher development initiatives.

**Methods:**

A nationally representative sample of 2,728 in-service PE teachers (1,758 males and 970 females) at the basic education level in China was recruited. Participants completed standardized scales assessing teaching motivation, teacher job burnout, and professional identity. Data were analyzed using SPSS 26.0 for reliability and validity assessments, exploratory factor analysis, group difference tests, and correlation analyses. Confirmatory factor analysis and structural equation modeling were conducted using Mplus 7.4 to examine model fit and mediating effects.

**Results:**

(1) The overall level of job burnout among female PE teachers remained below the clinical threshold; however, significant differences were observed across gender, educational stage, professional rank, teaching experience, and marital and parental status. Furthermore, scores across the dimensions of burnout exhibited notable variation. (2) Internal teaching motivation and professional identity were negatively associated with job burnout, whereas external teaching motivation demonstrated a positive association. (3) Mediation analysis revealed a significant indirect pathway—“intrinsic teaching motivation → professional identity → job burnout”—indicating partial mediation. In contrast, no such mediating pathway was found for extrinsic teaching motivation.

**Conclusion:**

Job burnout among female PE teachers in China’s basic education is generally within a manageable range, though significant gender and intragroup disparities exist. But emotional exhaustion emerges as a particularly salient concern. Internal and external teaching motivations exert distinct influences on burnout levels, with stronger internal motivation linked to reduced job burnout. Professional identity partially mediates the relationship between internal teaching motivation and job burnout. It is recommended that educational authorities prioritize fostering internal motivation and strengthening professional identity when designing interventions to mitigate teacher job burnout and support the professional growth of PE teachers.

## 1 Introduction

Burnout is a psychosocial syndrome characterized by prolonged exposure to excessive workload and chronic work-related stress, which can lead to the development of job burnout (1). As a global public health concern, “job burnout” refers to a set of psychological and behavioral manifestations experienced by individuals in the workplace that result in feelings of overwhelming burden, including long-term emotional exhaustion, physical fatigue, negative attitudes toward service recipients, and diminished job satisfaction (2). Due to the unique demands and inherent characteristics of teaching, educators are particularly vulnerable to burnout. Teacher burnout is specifically defined as a condition marked by emotional exhaustion, depersonalization, and reduced personal accomplishment (3). Empirical evidence further indicates that gender serves as a significant predictor of burnout risk, with women generally exhibiting higher levels of burnout compared to men (4). Consequently, as a critical risk factor, job burnout poses notable constraints on the professional development of female physical education teachers. Research has consistently demonstrated that prolonged burnout not only compromises teachers’ physical and mental well-being—manifesting in symptoms such as headaches, gastrointestinal disorders, depression, and anxiety (5)—but also leads to decreased job satisfaction and engagement (6), increased turnover intentions (7), and ultimately undermines students’ academic outcomes and overall teaching quality (8).

The onset of teacher burnout is influenced by an interplay of individual internal factors and external social environments. However, existing literature predominantly emphasizes the impact of external factors—such as perceived organizational support (9), principal leadership and support (10), and collegial collaboration (11)—while largely overlooking individual-level variables and their underlying mechanisms. In reality, for PE teachers, personal factors may play a more pivotal role in influencing burnout levels (12). This is because, relative to the complexity and limited controllability of external conditions, individual emotions and cognitive processes offer greater potential for intervention and practical applicability. Moreover, within China’s basic education system, female PE teachers represent a minority group, with significantly lower representation compared to their male counterparts (13). Their burnout experiences have received insufficient scholarly attention, despite facing elevated risks. Given that female PE teachers may encounter under-recognized burnout and its associated adverse consequences, this study focuses on two key individual factors—teaching motivation and professional identity—to examine their influence on job burnout among this population. In order to improve the psychological well-being of female PE teachers and ensure the sustainability of their career development, this study further aims to propose precise strategies to prevent and cope with job burnout, and to provide corresponding empirical support.

## 2 Literature review and hypotheses

### 2.1 Teachers’ motivation for teaching and job burnout

Teaching motivation refers to the psychological driving force that prompts an individual to enter and persist in the teaching profession (14). It constitutes a critical factor influencing women’s decision to pursue careers as physical education teachers, shaped not only by the intrinsic characteristics of the teaching profession but also by individuals’ personal and social experiences. In terms of its structure, teaching motivation is commonly conceptualized through the dual framework of internal and external motivation. For example, Watt et al. developed a two-dimensional model of teaching motivation grounded in expectancy-value theory (15). Internal motivation reflects an individual’s intrinsic valuation of teaching, including personal interest in the profession, perceived professional competence, a sense of accomplishment, and strong identification with the teaching role (16). In contrast, external motivation pertains to extrinsic rewards associated with the teaching profession, such as work-life balance, financial remuneration, job security, and occupational prestige (17).

Empirical studies have demonstrated a significant association between teaching motivation and teacher burnout (18). Specifically, when discrepancies arise between anticipated motivations and actual working conditions, teachers’ job satisfaction tends to decline, potentially leading to burnout or even attrition (19). Comparative analyses further suggest that, under similar working conditions, teachers with differing motivational orientations may exhibit varying susceptibility to burnout. Teachers with strong internal motivation generally demonstrate greater enthusiasm, enhanced perceptions of teaching efficacy, and stronger feelings of professional belonging; they may also mitigate burnout through positive teacher-student interactions (20). Conversely, those primarily driven by external motivations—such as salary and job stability—are more prone to experience burnout before attaining their desired outcomes (19).

Based on the above theoretical and empirical foundations, the following hypotheses are proposed:

*Hypothesis* 1: Internal teaching motivation has a significant negative effect on job burnout among female PE teachers.

*Hypothesis* 2: External teaching motivation has a significant positive effect on job burnout among female PE teachers.

### 2.2 Teachers’ motivation for teaching and professional identity

In accordance with social identity theory, individuals consistently endeavor to construct a favorable self-image and pursue social identity. Consequently, professional identity emerges as a pivotal psychological factor influencing the career development of PE teachers. Professional identity among teachers denotes “a positive attitude toward the teaching profession and a sense of identity with the teacher role, as well as the self-concept cultivated by teachers within the professional realm” (21). It serves as a reflection of the values, beliefs, and emotional attitudes that female PE teachers, as professional educators, hold toward their teaching endeavors (22). Teaching motivation significantly influences the degree of professional identity among teachers, with this impact commencing during the pre-service stage and extending into the post-service stage, continually shaping teachers’ professional cognition, affect, and judgment. As some studies have highlighted, the teaching motivation of normal university students is intricately linked to their professional identity and teacher efficacy, thereby influencing their future career choices (23). Teachers who opt to teach out of a genuine liking or passion for the teaching profession, that is, those whose internal teaching motivation predominates in their career selection, exhibit markedly higher scores in professional identity and self-efficacy compared to other groups (24). This perspective is further corroborated by another cross-sectional survey conducted among young social workers in China, which posited a correlation between diverse career motivations and the professional identity of young social workers. Notably, young social workers driven by internal motivation and composite motivation demonstrated higher levels of professional identity (25).

Based on the above theoretical and empirical foundations, the following hypotheses are proposed:

*Hypothesis* 3: Internal teaching motivation has a significant positive effect on the professional identity of female PE teachers.

*Hypothesis* 4: External teaching motivation has a significant negative effect on the professional identity of female PE teachers.

### 2.3 The mediating role of professional identity

A higher level of professional identity signifies that teachers possess more affirmative professional cognition, emotions, and behaviors, which constitute a stable and constructive individual psychological resource capable of offering safeguards for teachers’ career advancement. For instance, a cross-sectional survey conducted among basic education teachers in China revealed that teachers’ professional identity contributed to enhancing work engagement and career satisfaction, thereby effectively mitigating job burnout (26). Indeed, in contrast to the transient impacts engendered by external factors such as salary, benefits, and bonuses, professional identity, as an enduring intrinsic factor, exerts a more pronounced effect in alleviating teacher burnout over the long term (27).

Based on the above theoretical and empirical foundations, the following hypotheses are proposed:

*Hypothesis* 5: Professional identity significantly negatively affects burnout in female PE teachers.

Teaching motivation exerts not only a direct influence on teachers’ job burnout but also an indirect impact mediated through various variables, including a sense of belonging, a sense of mission, personality traits, and so forth. In accordance with the Job Demand-Resource (JD-R) model’s explication, job characteristics encompass both job resources and job demands, which initiate distinct processes and pathways (28). Job demands denote factors at psychological, physiological, and organizational levels that necessitate individuals’ sustained efforts in their work, encompassing emotional management, role stress, physical condition, and work tasks. These demands deplete individuals’ physical and psychological resources, culminating in job burnout, they are called pressure processes or depletion paths. Conversely, job resources refer to psychological, physical, and organizational factors within the work environment that offer support to individuals, such as job autonomy, social support, and remuneration. These resources foster increased work engagement and yield more positive outcomes, a phenomenon termed the motivational process or gain path (29). A plethora of studies grounded in the JD-R model corroborate that job demands positively predict job burnout, whereas job resources negatively predict it (30). This implies that professional identity, as a pivotal job resource for teachers, can bolster the teaching motivation of female PE teachers, propelling them to heighten their enthusiasm and commitment to teaching endeavors. Consequently, this leads to more favorable outcomes, such as professional fulfillment and a thriving sense of work, while concurrently curbing the accumulation of negative emotions associated with job burnout.

Based on the above theoretical and empirical foundations, the following hypotheses are proposed:

*Hypothesis* 6: Professional identity of female PE teachers serves as a mediating factor in the influence of internal teaching motivation on job burnout.

*Hypothesis* 7: Professional identity of female PE teachers serves as a mediating factor in the influence of external teaching motivation on job burnout.

## 3 Methods

### 3.1 Sample

The stratified random sampling approach was employed to survey in-service PE teachers at the basic education across mainland China, with special schools, vocational schools, and regions of Hong Kong, Macao, and Taiwan excluded from the study. From May 1 to May 20, 2025, a total of 2,836 questionnaires were administered online, all of which were returned. After excluding 108 invalid responses, 2,728 valid questionnaires were retained, yielding an effective response rate of 96.2%.

Among the valid responses, 970 were from female PE teachers, representing 35.6%, while 1,758 were from male PE teachers, accounting for 64.4%, a distribution consistent with the demographic characteristics of PE teachers in China. Regarding educational attainment, 46 teachers held a college degree or below, comprising 1.69% of the sample; 2,036 teachers possessed a bachelor’s degree, accounting for 74.63%; and 647 teachers had a master’s degree or higher, representing 23.71%. In terms of professional titles, 682 teachers held third-level or unrated titles, making up 24.90%; 872 teachers had second-level titles, accounting for 31.96%; 873 teachers possessed first-level titles, representing 32.00%; and 300 teachers held senior titles, comprising 10.10%. Regarding teaching experience, 1,173 teachers had 5 years or less of teaching experience, accounting for 43.0%; 545 teachers had 6 to 10 years of experience, representing 20.0%; 409 teachers had 11 to 15 years of experience, making up 15.0%; 109 teachers had 16 to 20 years of experience, accounting for 4.0%; and 492 teachers had 21 years or more of experience, representing 18.0%. In terms of the educational institution type, 1,445 teachers were from primary schools, accounting for 52.9%; 846 teachers were from junior high schools, representing 31.0%; and 464 teachers were from senior high schools, making up 17.0%. Regarding monthly income, 272 teachers earned less than 3,000 RMB, accounting for 10.0%; 818 teachers earned between 3,000 and 4,499 RMB, representing 30.0%; another 818 teachers earned between 4,500 and 5,999 RMB, also accounting for 30.0%; and 464 teachers earned between 6,000 and 7,499 RMB, making up 17.0%. Additionally, 218 teachers had a monthly income ranging from 7,500 to 8,999 RMB, accounting for 8.0%, and 138 teachers earned more than 9,000 RMB per month, representing 5%. In terms of marital status, 846 teachers were unmarried, accounting for 31%; 272 teachers were married but childless, representing 10%; and 1,610 teachers were married and had children, making up 59% of the sample.

### 3.2 Data collection tools

The questionnaire was structured into four distinct sections: Basic Information, Professional Identity, Job Burnout, and Teaching Motivation.

(1) The Basic Information section encompassed a range of demographic and professional attributes of PE teachers, including gender, educational background, teaching tenure, professional title, duration of formal education, salary level, and marital and reproductive status.
(2) In the Professional Identity section, the Professional Identity Scale for Primary and Secondary School Teachers, developed by Wei Shuhua, was employed. This scale comprised four dimensions: role values, professional values, professional sense of belonging, and professional behavior tendency (31), with a total of 18 items. A Likert 5-point scoring system was utilized, ranging from “completely inconsistent” to “completely consistent,” with corresponding scores of 1 to 5. Higher scores indicated a greater degree of professional identity among PE teachers. The overall Cronbach’s *α* coefficient for the scale was 0.91>0.7, thus demonstrating excellent reliability. The KMO = 0.87>0.6, indicating good validity. We employed the MLR estimator for the confirmatory factor analysis, the model fit indices were as follows: χ^2^/df = 2.507, RMSEA = 0.067, CFI = 0.953, TLI = 0.935, and SRMR = 0.052.
(3) For the Job Burnout section, the MBI-ES, originally compiled by Maslach and Jackson (32), and the Job Burnout Questionnaire for Chinese Primary and Secondary School Teachers, revised by Chinese scholar Wu Xinchun (33), were referenced. This section contained 22 items distributed across three dimensions: emotional exhaustion, personal accomplishment, and depersonalization. A Likert 5-point scoring method was applied, with scores ranging from 1 (“never”) to 5 (“always”). Higher scores indicated a stronger sense of job burnout. The total Cronbach’s *α* = 0.91>0.7, indicating excellent reliability, while the KMO = 0.85>0.6, suggesting good validity. We employed the MLR estimator for the confirmatory factor analysis, the model fit indices were χ^2^/df = 3.286, RMSEA = 0.071, CFI = 0.914, TLI = 0.902, and SRMR = 0.065.
(4) In the Teaching Motivation section, the teacher motivation scale from the TALIS 2018 teacher questionnaire was adapted for use, encompassing two dimensions: internal motivation and external motivation (34), with a total of seven items. A Likert 4-point scale was employed, with scores ranging from 1 (“not at all important”) to 4 (“very important”). Higher scores indicated stronger internal or external motivation. The total Cronbach’s *α* = 0.79>0.7, indicating good reliability, while the KMO = 0.85>0.6, suggesting good validity. We employed the MLR estimator for the confirmatory factor analysis, the model fit indices were χ^2^/df = 4.043, RMSEA = 0.053, CFI = 0.991, TLI = 0.957, and SRMR = 0.028.

### 3.3 Statistical analysis

SPSS 26.0 software was used for reliability analysis, validity analysis, exploratory factor analysis, difference test and correlation analysis. Mplus 7.4 was used for confirmatory factor analysis and structural equation model analysis.

## 4 Results

### 4.1 Common method bias testing

Because this study used self-reported reporting, the presence of serious common methods bias needed to be confirmed. The results were analyzed by Harman single factor test, and the results showed that there were a total of 5 factors with characteristic roots greater than 1. The first factor only explained 23.09% of the variance, which was less than the critical standard of 40%, so the influence of common method bias was not significant.

### 4.2 Descriptive statistics and correlation analysis

As shown in Table 1, female PE teachers in China’s basic education are dominated by external teaching motivation and have a high degree of professional identity, but their job burnout is at a moderate level and emotional exhaustion is relatively prominent. Through further difference analysis, it was found that:

(1) There are significant gender differences in job burnout and teaching motivation of PE teachers, but there is no significant gender difference in professional identity. On the one hand, the overall level of job burnout of female PE teachers (M = 2.46) was higher than that of male PE teachers (M = 2.41), and the difference was significant (*t* = 2.36, *p* < 0.05). On the other hand, female and male PE teachers also showed some differences in various dimensions of job burnout, professional identity and motivation to teach. ① In terms of job burnout, there were significant differences in the three dimensions of job burnout between female and male PE teachers. The scores of emotional exhaustion (M = 3.00) and low personal accomplishment (M = 2.41) in female group were significantly higher than those in male group (*t* = 2.03, *p* < 0.05; t = 4.78, *p* < 0.01), while the score of male teachers in the dimension of dehumanization (M = 2.21) was significantly higher than that of female teachers (*t* = −3.28, *p* < 0.01). ② In terms of professional identity, the scores of professional belonging and professional behavior tendency of female PE teachers were higher than those of male PE teachers, the scores of role values and professional values of male PE teachers were slightly higher than those of female PE teachers, but only the differences of professional values and professional behavior tendency among the four dimensions had very significant gender differences (*t* = 3.727, *p* < 0.01; *t* = 3.503, *p* < 0.01). ③ In terms of teaching motivation, the score of internal motivation of female PE teachers (M = 3.05) was lower than that of male PE teachers (M = 3.27), and the difference was statistically significant (*t* = −6.28, *p* < 0.01), but there was no significant gender difference in external motivation. This result may be attributed to gender stereotypes within the field of sports. On the one hand, physical education has long been constructed as a domain characterized by “masculinity,” emphasizing strength, competition, and authority. This occupational gender segregation affords male teachers a higher level of Person-Environment Fit; they are more likely to view physical education as a pathway for self-actualization, thereby eliciting stronger intrinsic motivation. On the other hand, stereotype threat may suppress the motivational experience of female teachers. In a male-dominated sports culture, female teachers may face implicit pressure regarding “competence doubts” or be required to engage in greater emotional labor to adapt to non-traditional gender role expectations. Such psychological depletion diminishes their sense of enjoyment and competence derived from the teaching activity itself.
(2) There are significant differences in job burnout and professional identity among female PE teachers, but there is no significant difference in teaching motivation. ① There are significant differences in job burnout among female PE teachers with different teaching years, school period and salary level. For example, there was a significant difference between the different groups of teachers (*F* = 11.05, *p* < 0.01), especially the female physical education teachers in primary school had a more prominent phenomenon of job burnout, and their emotional exhaustion scores were higher (M = 3.16). This phenomenon may be related to the psychological development characteristics and emotional needs of the students in different grades. Due to the low psychological maturity of primary school students, they have a strong emotional dependence on physical education teachers and need more emotional care from teachers, while junior and senior high school students have increased self-awareness and independence, and their emotional needs for physical education teachers are reduced, and the intensity of teachers’ emotional labor is reduced, making primary school teachers more prone to emotional exhaustion. For example, the score of emotional exhaustion in the married and childbearing group was significantly higher than that in the unmarried and married without childbearing group (*F* = 3.55, *p* < 0.05). This may be related to the maternal role of women; female PE teachers who had children faced more work–family conflicts than the other groups, and emotional labor was a very important source of stress. Whether as mothers or teachers, they need to continuously output a large number of invisible emotions in different time and space, so the level of emotional exhaustion is higher.

**Table 1:** Descriptive statistics and correlation analysis of main variables (*N* = 970).

Variables	Mean	SD	1	2	3	4
1. External teaching motivation	3.32	0.57	1			
2. Internal teaching motivation	3.05	0.63	0.45**	1		
3. Professional identity	4.12	0.66	0.19**	0.47**	1	
4. Job burnout	2.46	0.56	−0.15*	−0.38**	−0.55**	1

*p < 0.05, **p < 0.01.

② There are significant differences in the total professional identity of female PE teachers with different teaching years, professional titles, marriage and childbearing status, school period and salary level. For example, the identification degree of senior teachers and first-level teachers on the PE teaching profession was significantly higher than that of teachers with second-level and below professional titles (*F* = 6.01, *p* < 0.01), and the scores of each identity dimension also showed similar distribution rules. Female PE teachers with different educational background only had significant differences in the dimension of role values (*t* = −1.970, *p* < 0.05), and there was an “inverted phenomenon” that the score of teachers with bachelor degree was higher than that of teachers with master degree. The reason for this phenomenon may be related to the career expectations of different groups. Physical education teachers usually have higher career expectations and tend to set higher development goals and work standards in practice.

(3) According to the results of correlation analysis in Table 1, it can be seen that there is a significant negative correlation between the external motivation for teaching, the internal motivation for teaching, the professional identity and the job burnout of female PE teachers. From the specific correlation coefficient, the correlation between professional identity and job burnout of female PE teachers was high, and the correlation coefficient was −0.55. The correlation coefficients between external motivation and internal motivation, internal motivation and professional identity of female PE teachers were all over 0.4, indicating that there was a moderate correlation between the two groups of variables. In addition, there were low correlations between job burnout and external motivation to teach, professional identity and external motivation to teach, and professional identity and internal motivation to teach.

### 4.3 The influence of different teaching motivations on job burnout

To ascertain whether internal and external motivation exert a statistically significant impact on job burnout among female PE teachers, a direct effect test was conducted. The statistical outcomes revealed that the model fit indices were as follows: χ^2^/df = 4.528, RMSEA = 0.052, CFI = 0.966, TLI = 0.959, and SRMR = 0.055. Based on the standardized regression coefficients derived from the model, it was observed that internal motivation among female PE teachers demonstrated a highly significant negative effect on their job burnout, as evidenced by a coefficient of *β* = −0.215 (*p* < 0.01). Conversely, external motivation exhibited a highly significant positive effect on job burnout among female PE teachers, with a standardized regression coefficient of *β* = 0.373 (*p* < 0.01). While internal motivation was associated with a reduction in burnout, this finding suggests that higher levels of external motivation are associated with an increase in teacher burnout. These results provide compelling evidence for the differential impacts of internal and external motivation on job burnout among female PE teachers, highlighting the importance of considering both types of motivation in efforts to mitigate burnout.

### 4.4 Test of the mediating role of professional identity

To further investigate the mediating role of professional identity in the relationship between teaching motivation and job burnout among female physical education (PE) teachers, a Bootstrap analysis with bias-corrected non-parametric percentiles was employed. This approach involved resampling the dataset 2,000 times to ensure robust estimation of the mediating effects. Because Bootstrapping does not rely on distributional assumptions and provides bias-corrected confidence intervals (CI), which is considered the gold standard for testing mediation effects (35). The statistical outcomes of this analysis are detailed in Table 2. The model fit indices derived from the structural equation modeling were as follows: χ^2^/df = 4.033, RMSEA = 0.057, CFI = 0.952, TLI = 0.903, and SRMR = 0.071. In accordance with established methodological guidelines (36), the presence or absence of a significant mediating effect was determined by examining whether the 95% confidence interval (CI) of the estimated mediating effect included zero. Specifically, a mediating effect was deemed significant if the 95% CI did not encompass zero. Notably, the three variables under investigation collectively accounted for 38.5% of the variance in job burnout within this demographic.

**Table 2:** Test of the mediating effect of professional identity.

Effect	Estimate	SE	Z	95% CI	Sig.
External motivation for teaching → Professional identity → Job burnout					
Total effect	0.147	0.054	2.722	[0.042,0.203]	0.00
Indirect effect	0.032	0.028	1.143	[−0.022,0.068]	0.57
Direct effect	0.115	0.035	3.286	[0.038,0.188]	0.00
Internal motivation for teaching → Professional identity → Job burnout					
Total effect	−0.443	0.040	−10.825	[−0.561,-0.329]	0.00
Indirect effect	−0.247	0.058	−4.259	[−0.408,−0.186]	0.00
Direct effect	-0.186	0.044	−4.227	[−0.284,-0.101]	0.00

**Figure 1:**
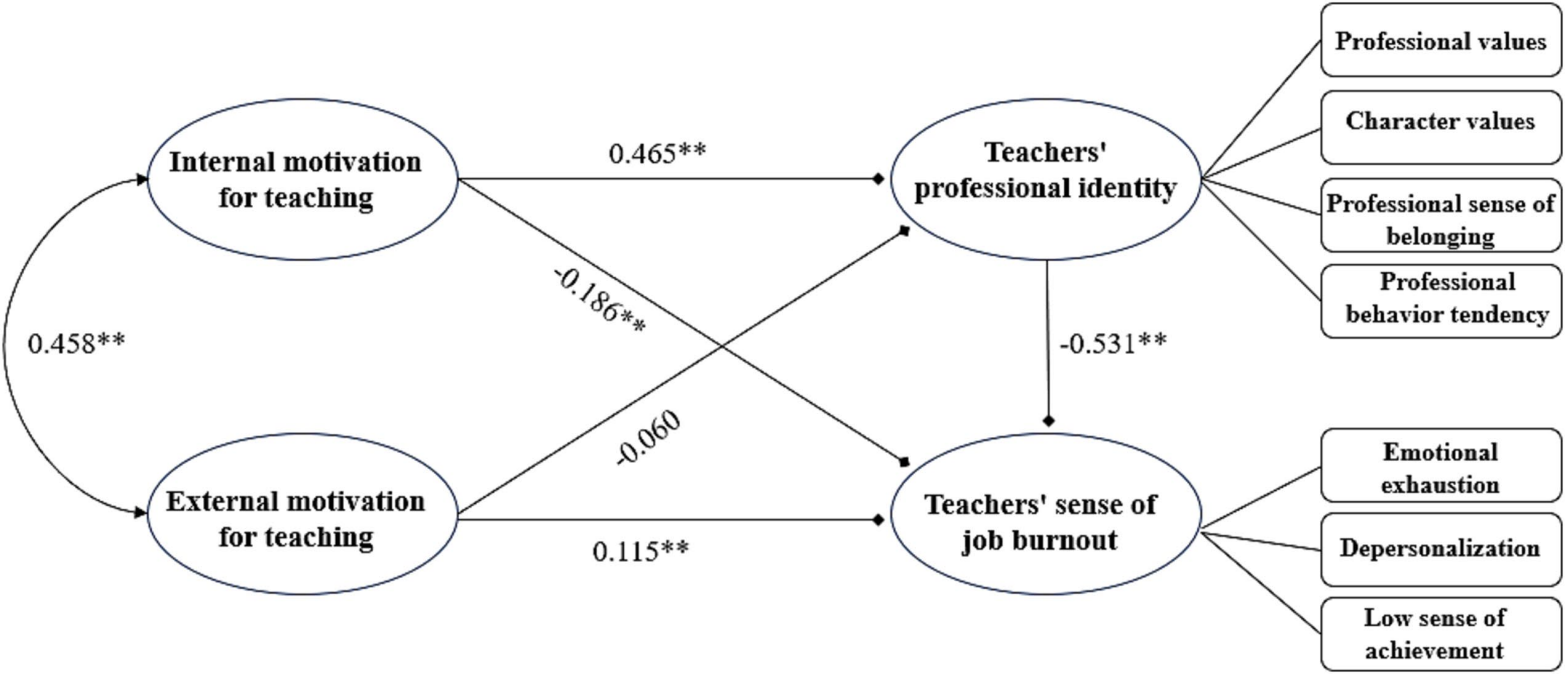
Model diagram of the influence of teaching motivation and professional identity on job burnout in female PE teachers. **means *p* < 0.01.

Combining Figure 1 and Table 2, the following conclusions can be drawn: (1) Professional identity of female PE teachers has a negative and significant effect on teachers’ job burnout (*β* = −0.531, *p* < 0.01). (2) The total effect of external motivation of female PE teachers on teachers’ job burnout was significant (*β* = 0.147, CI[0.042, 0.203], *p* < 0.01), and the external motivation of female PE teachers had a positive and significant effect on teachers’ job burnout (*β* = 0.115, CI[0.038, 0.188] *p* < 0.01; 0.188, *p* < 0.01). The mediating effect of teachers’ professional identity on the influence of external motivation to teach on teachers’ job burnout was not established (*β* = 0.032, CI[−0.022, 0.068], *p* > 0.05). (3) The total effect of internal motivation of female PE teachers on teachers’ job burnout was significant (*β* = −0.443, CI[−0.561, 0.329], *p* < 0.01), among which the internal motivation of female PE teachers had a negative and significant effect on teachers’ job burnout (*β* = −0.186, CI[−0.284, −0.101], *p* < 0.01). The mediating effect of teachers’ professional identity on the influence of teaching motivation on teachers’ job burnout was established (*β* = −0.247, CI[−0.408, −0.186], *p* < 0.01).

## 5 Discussion

### 5.1 The overall characteristics of job burnout among female PE teachers

(1) The level of job burnout among female PE teachers in China’s basic education system is generally within a manageable range. Both the overall burnout scores and the results of subgroup comparisons indicate that job burnout in this group has not exceeded the critical threshold. This suggests not only that these teachers are not experiencing an excessively high subjective workload, but also creates favorable conditions for government-led initiatives to reduce teacher burden. Furthermore, it supports individual teachers’ mental well-being and professional development. This finding aligns with a longitudinal study on job burnout among primary and secondary school teachers in China (4), suggesting that, compared to teachers in other subject areas, PE teachers do not exhibit discipline-specific patterns of burnout.
(2) Significant gender differences and intra-group variations exist in the levels of job burnout among female PE teachers in China. On one hand, female PE teachers report significantly higher burnout scores than their male counterparts—a result consistent with existing literature indicating that female teachers generally experience higher levels of burnout (6). On the other hand, notable differences emerge across subgroups of female PE teachers based on teaching experience, educational stage (e.g., primary vs. secondary), and salary level. In particular, emotional exhaustion is more pronounced among those teaching at the primary school level, which contrasts with findings from other studies suggesting that secondary school teachers typically exhibit the highest burnout levels (37). This discrepancy may be attributed to the unique characteristics of PE instruction, including the greater emotional care demands associated with younger students, resulting in increased emotional labor intensity. Moreover, current policy in Chinese primary schools mandates “one PE class per day,” whereas middle and high schools allocate fewer weekly PE hours due to academic pressures. Consequently, primary school PE teachers often face heavier workloads and higher job intensity, placing them at significantly greater risk for both job burnout and emotional exhaustion compared to their peers in later educational stages.
(3) Female PE teachers in China demonstrate relatively high levels of personal accomplishment and humanization; however, emotional exhaustion remains a salient concern. This dimensional distribution pattern is consistent with findings reported by other Chinese researchers. As shown in the comparative analysis, emotional exhaustion exceeds the critical value—particularly among primary school teachers and those who are married with children. According to Maslach and Schaufeli, the three dimensions of job burnout are interrelated and potentially causal (38). Numerous studies—including those by Leiter and Maslach (39), Lee and Ashforth (40), and Taris (41)—have identified emotional exhaustion as the initial and most predictive dimension of burnout development. Therefore, elevated emotional exhaustion may signal an increased likelihood of overall burnout in the future, warranting closer attention from relevant governmental and educational authorities. In contrast, lower scores in depersonalization and reduced personal accomplishment suggest that this group currently maintains a relatively positive professional attitude and adaptive coping strategies. This may be influenced by China’s cultural emphasis on “respecting teachers and valuing education” as well as ongoing efforts to strengthen teacher ethics and professional conduct.

### 5.2 The impact of teaching motivation on job burnout among female PE teachers

Both external motivation and internal motivation have positive and negative significant effects on job burnout of female PE teachers, respectively, indicating that hypotheses 1 and 2 have been verified. Because there is a close relationship between teachers’ cognition of the profession and their career motivation to choose teachers (42), this means that when female PE teachers enter the profession because they like students, like teaching and are willing to contribute to the development of education, they are more likely to participate in physical education activities with a positive attitude and have a lower level of job burnout. When female PE teachers choose to be teachers because they desire a stable and decent career, enjoy a long vacation and more social welfare, they are more likely to feel job burnout when the expected return does not match the effort in work, and then choose to leave or change careers, which is consistent with the results of previous studies (43). Furthermore, this conclusion directly aligns with the core tenets of the JD-R model. As a job resource for female PE teachers, intrinsic teaching motivation not only negatively predicts burnout in this group but also fosters their work engagement, whereas extrinsic teaching motivation failed to yield similar effects.

In this regard, it is suggested that individuals should fully examine whether they have the internal motivation to become a PE teacher before choosing a career, which is more conducive to the sustainable development of the teacher career in the future. In the process of training pre-service physical education teachers or recruiting talents, schools and education management departments also need to investigate the teaching motivation of students or applicants, and screen out more teachers with strong internal teaching motivation, which is more conducive to maintaining the stability of the physical education teachers.

### 5.3 The mediating role of professional identity between teaching motivation and job burnout among female PE teachers

The professional identity of female PE teachers has a significant negative effect on job burnout, and professional identity plays a partial mediating role in the influence of teaching motivation on job burnout in female PE teachers.

Firstly, professional identity of female PE teachers significantly negatively affected job burnout. This study confirmed that the professional identity of teachers significantly negatively affects their job burnout, and hypothesis 5 was verified, which is consistent with the conclusions of previous studies (26, 44). This shows that professional identity is the final result of teachers’ professional cognition and professional emotion, which will have a key impact on individual teachers’ physical and mental health and career development, can inhibit or reduce teachers’ job burnout. Female teachers in China often face a “double burden” of professional expectations and traditional family responsibilities. As career success becomes a primary source of self-worth and social status, potentially making them more resilient to burnout when their professional identity is affirmed. Additionally, consistent with the assumptions of the JD-R model, professional identity can be conceptualized as a job resource for female physical education teachers, one that is capable of generating a corresponding gain process. Enhancing teachers’ professional identity should be the consensus of individual female PE teachers, school administrators and local education management departments.

Secondly, the professional identity of female PE teachers played a partial mediating role in the internal effect of teaching motivation on job burnout. The results from the mediation effect test showed that: in the influence of internal teaching motivation on job burnout of female PE teachers, there was an influence path of “internal teaching motivation → professional identity → job burnout,” but in the influence of external teaching motivation on job burnout of female PE teachers, there was no influence path of “external teaching motivation → professional identity → job burnout.” That is, Hypothesis 6 is verified and hypothesis 7 is not. Among the influence of female physical education teachers’ internal motivation on teachers’ job burnout, the indirect effect of professional identity accounted for 58.6%, and the direct effect accounted for 41.4%, which showed that the influence of female PE teachers’ internal motivation on teachers’ job burnout was mostly affected by professional identity. Therefore, it is believed that enhancing the internal motivation and professional identity of female PE teachers is of great value to inhibit or reduce the job burnout of this group.

### 5.4 Limitations

Although this study quantitatively evaluated the job burnout and motivation of female physical education teachers in basic education in China through questionnaire survey, and verified the mediating role of professional identity between the two variables, there are still some limitations in this study that need to be improved in future research.

Firstly, this study only adopts a cross-sectional design, which can only reveal the current job burnout and professional identity of the respondents, and cannot further explore and track the causal mechanism and dynamic changes of job burnout of Chinese female PE teachers.

Secondly, only the in-service PE teachers in ordinary primary and secondary schools were investigated, and more groups were not included in the comparative analysis, such as female PE teachers from special schools, vocational schools, other countries and universities, which limited the scope of the applicable population of the research conclusions.

Thirdly, this study did not control for variables such as school level, teaching experience, and marital and childbearing status during the direct and mediation effect analyses. Consequently, we were unable to fully isolate the ‘pure effects’ of motivation for teaching and professional identity on the job burnout of female PE teachers. Future research should incorporate these variables as covariates into more complex SEM models (e.g., multi-group SEM).

Finally, this study mainly focused on the influence of individual level factors on the job burnout of female PE teachers, and did not include external factors such as organization and environment, resulting in certain deficiencies. For example, for female PE teachers who are married and have children, parenting stress in the home environment may cause work–family conflict and further translate into a source of stress that exacerbates their job burnout.

## 6 Conclusion

First, the level of job burnout among female PE teachers in China’s basic education system is generally manageable, yet notable differences exist in terms of gender, intra-group characteristics, and specific dimensions of burnout. Female PE teachers exhibit a slightly higher level of job burnout compared to their male counterparts. In particular, emotional exhaustion is more pronounced among female PE teachers in primary schools and those who are married with children, which may be attributed to gender-specific social roles, students’ emotional demands, and the division of labor within both professional and family contexts. Second, the internal and external motivation of female PE teachers had significant negative and positive effects on job burnout, respectively. Specifically, stronger internal motivation is linked to lower levels of burnout, whereas greater reliance on external motivation increases the risk of burnout. Third, professional identity serves as a partial mediator in the relationship between teaching motivation and job burnout among female PE teachers.

Finally, given the multifaceted nature of job burnout in this group, it is recommended that educational authorities and school administrators acknowledge the complexity of teaching work and adopt individualized feedback and intervention strategies in managing teacher well-being. Particularly in the context of PE teacher training, greater emphasis should be placed on fostering internal motivation and strengthening professional identity. For instance, it is advisable to establish female role models by showcasing cases of outstanding female physical education teachers, thereby demonstrating diverse career success paths. Through the regular organization of various activities, teachers can be encouraged to share their experiences regarding professional dilemmas, emotional management, and work-family balance. Additionally, policies should mandate gender-sensitive support systems, such as flexible working arrangements for female teachers balancing family and career.

## Data Availability

The original contributions presented in the study are included in the article/supplementary material, further inquiries can be directed to the corresponding author.
